# A new monoclonal antibody detects downregulation of protein tyrosine phosphatase receptor type γ in chronic myeloid leukemia patients

**DOI:** 10.1186/s13045-017-0494-z

**Published:** 2017-06-21

**Authors:** Marzia Vezzalini, Andrea Mafficini, Luisa Tomasello, Erika Lorenzetto, Elisabetta Moratti, Zeno Fiorini, Tessa L. Holyoake, Francesca Pellicano, Mauro Krampera, Cristina Tecchio, Mohamed Yassin, Nader Al-Dewik, Mohamed A. Ismail, Ali Al Sayab, Maria Monne, Claudio Sorio

**Affiliations:** 1 Department of Medicine, University of Verona, Strada le Grazie 8, 37134 Verona, Italy; 2 ARC-Net Research Centre, University and Hospital Trust of Verona, 37134 Verona, Italy; 3 Section of Physiology, Department of Neurological, Neuropsychological, Morphological and Motor Sciences, University of Verona, Verona, Italy; 4 Paul O’Gorman Leukaemia Research Centre, College of Medical, Veterinary & Life Sciences, Institute of Cancer Sciences, University of Glasgow, Glasgow, UK; 5 The Beatson Institute for Cancer Research, Garscube Estate, Switchback Road, Bearsden, Glasgow, G61 1BD Scotland UK; 6 Section of Hematology, Department of Medicine, University of Verona, Verona, Italy; 7 National Center for Cancer Care and Research (NCCCR), Hamad Medical Corporation (HMC), Doha, Qatar; 8 Qatar Medical Genetics Center, Hamad Medical Corporation (HMC), Doha, Qatar; 9 Interim Translational Research Institute (iTRI), Hamad Medical Corporation, Doha, Qatar; 10 Centro di Diagnostica Biomolecolare e Citogenetica Emato-Oncologica, “San Francesco” Hospital, ASL3, Nuoro, 08100 Italy; 11 Present address: The Ohio State University, Wexner Medical Center Biomedical Research Tower, 460W 12th Avenue, room 1070, Columbus, OH 43210 USA

**Keywords:** Monoclonal antibody, Protein tyrosine phosphatase, Chronic myeloid leukemia, Tumor suppressor gene, BCR-ABL1

## Abstract

**Background:**

Protein tyrosine phosphatase receptor gamma (PTPRG) is a ubiquitously expressed member of the protein tyrosine phosphatase family known to act as a tumor suppressor gene in many different neoplasms with mechanisms of inactivation including mutations and methylation of CpG islands in the promoter region. Although a critical role in human hematopoiesis and an oncosuppressor role in chronic myeloid leukemia (CML) have been reported, only one polyclonal antibody (named chPTPRG) has been described as capable of recognizing the native antigen of this phosphatase by flow cytometry. Protein biomarkers of CML have not yet found applications in the clinic, and in this study, we have analyzed a group of newly diagnosed CML patients before and after treatment. The aim of this work was to characterize and exploit a newly developed murine monoclonal antibody specific for the PTPRG extracellular domain (named TPγ B9-2) to better define PTPRG protein downregulation in CML patients.

**Methods:**

TPγ B9-2 specifically recognizes PTPRG (both human and murine) by flow cytometry, western blotting, immunoprecipitation, and immunohistochemistry.

**Results:**

Co-localization experiments performed with both anti-PTPRG antibodies identified the presence of isoforms and confirmed protein downregulation at diagnosis in the Philadelphia-positive myeloid lineage (including CD34^+^/CD38^bright/dim^ cells). After effective tyrosine kinase inhibitor (TKI) treatment, its expression recovered in tandem with the return of Philadelphia-negative hematopoiesis. Of note, PTPRG mRNA levels remain unchanged in tyrosine kinase inhibitors (TKI) non-responder patients, confirming that downregulation selectively occurs in primary CML cells.

**Conclusions:**

The availability of this unique antibody permits its evaluation for clinical application including the support for diagnosis and follow-up of these disorders. Evaluation of PTPRG as a potential therapeutic target is also facilitated by the availability of a specific reagent capable to specifically detect its target in various experimental conditions.

**Electronic supplementary material:**

The online version of this article (doi:10.1186/s13045-017-0494-z) contains supplementary material, which is available to authorized users.

## Background

Protein kinases and phosphatases act in a coordinated manner enabling precise regulation of signaling responses [1]. Protein tyrosine phosphatase receptor gamma (PTPRG) belongs to subtype V of the receptor-type protein tyrosine phosphatases (PTPs) and possesses an extracellular region, a single transmembrane region, and two tandem intracytoplasmic catalytic domains. Membranous and/or cytoplasmic PTPRG expression has been detected in leukocytes, including hematopoietic precursors, the vast majority of epithelial and endocrine cells of various organs [2–4]. *PTPRG* is a candidate tumor suppressor gene, and loss of function by mutation, deletion, or loss of heterozygosity have been associated with different types of tumors [5–7].

We have previously demonstrated that methylation-driven downregulation of *PTPRG* expression occurs in chronic myeloid leukemia (CML). Forced overexpression of PTPRG led to reduced tyrosine phosphorylation, decreased clonogenic capability, and induction of apoptosis in CML cells. In the same study, we have shown that BCR-ABL1, the key deregulated kinase in CML, is a biochemical substrate for PTPRG [8]. Two epitope-specific antisera against the extracellular domain (ECD) of PTPRG have previously been developed from rabbit [9] and chicken [10]. However, polyclonal antibodies do not represent an unlimited source of antibody, and variability in the preparation and batch might affect specificity, affinity, and avidity toward the antigen. Conversely, monoclonal antibodies represent virtually unlimited and homogeneous sources of reagents, as they are derived from immortalized cells (hybridomas) and have applications in virtually all areas of biology and medicine. Early diagnosis and cure for patients with CML represent significant clinical challenges because of disease progression from chronic phase (CP) into a rapidly fatal blast crisis (BC). Few protein biomarkers have been described and implemented for CML diagnosis or management, most are overexpressed, often not CML specific and, overall, need to be better characterized and validated in the clinic [11–14]. An exception to this general picture might be CD26 and IL1RAP that were very recently described as a CD34^+^/CD38^−^ CML leukemia stem cell (LSC)-associated biomarker [15–17]. Overall cell surface antigens deserve more in-depth characterization as they have the potential to represent a complementary, robust, and straightforward method for monitoring the disease and may represent potential therapeutic targets [11–13].

In the present study, we describe a new monoclonal antibody developed against the ECD of human PTPRG. The antibody, named TPγ B9-2, was found to react against both human and murine PTPRG and was tested for immunoblotting, immunoprecipitation, immunohistochemistry, and flow cytometry applications. Its versatility across a wide range of applications validates this new antibody as a critical advance and a powerful tool to provide a more detailed characterization of the tumor suppressor gene PTPRG in the context of human and murine cells and tissues, including evaluation of clinical samples.

## Methods

Additional procedures are described in detail in the Additional file 1.

### Production of anti-PTPRG monoclonal antibody

Mouse hybridoma cell lines were produced by ARETA International srl (Gerenzano VA, Italy) from BALB-C mice that were challenged with the purified ECD of human PTPRG (ENST00000295874, NM_002841.3). The ECD complementary DNA (cDNA) was cloned in a pCEP4 vector containing an in-frame, carboxy-terminal murine IgG_3_-Fc portion used as a tag for purification purposes. The vector was kindly provided by Dr. Maria Laura Belladonna, University of Perugia, Italy. The construct obtained, named pCEP4-Fc-ECD, was transduced into the HEK293F human embryonic kidney cell line. The cells were cultured in protein-free medium CD293® (Thermo Fisher, Milan, Italy) with 50 μM β-mercaptoethanol and 0.5 mg/mL of hygromycin (Thermo Fisher, Milan, Italy). Conditioned medium was collected, and fusion protein was purified (>95%) by protein-G affinity chromatography. Individual hybridoma clones were first evaluated by direct ELISA assay using the purified ECD, the Fc portion of which was cleaved off to identify and remove all the clones recognizing the tag.

Supernatants from individual subclones were screened on the basis of capability to recognize the native antigen expressed on the surface of peripheral blood monocytes by flow cytometry. After a further round of subcloning from a positive candidate, the TPγ B9-2 clone (IgG_1_) was selected.

### Primary cell samples

Fresh leukapheresis or peripheral blood samples were obtained with written informed consent from patients with CP CML at diagnosis prior to treatment or non-CML donors. We have determined the expression levels of *PTPRG* by real-time quantitative polymerase chain reaction (RQ-PCR) in peripheral blood in 39 CML patients at presentation and after treatment with tyrosine kinase inhibitors (TKIs) (Additional file 1: Table S2). CML patients included in this study were diagnosed in CP at the San Francesco Hospital, Nuoro, and at the National Center for Cancer Care and Research (NCCCR), Hamad Medical Corporation (HMC), Doha-Qatar. The study was approved by the Local Ethics Committee, ASL Nuoro (Permit Number: 1276), AOUI Verona (Permit Number: 25066), and Hamad Medical Center, Doha, Qatar (HMC Permit Number: 2011/1118). Informed consent in accordance with the declaration of Helsinki was obtained from each patient. Fresh samples used for flow cytometry (24 CML and 13 G-CSF mobilized individuals, mean age 30, range 11–53, eight males, five females) were used as whole blood or, in selected cases, were enriched for CD34^+^ cells using CliniMACS (Miltenyi Biotec Inc., Auburn, CA, USA) according to the manufacturer’s instructions or as previously reported [18].

## Results

### Immunoprecipitation and western blotting analysis

**Fig. 1:**
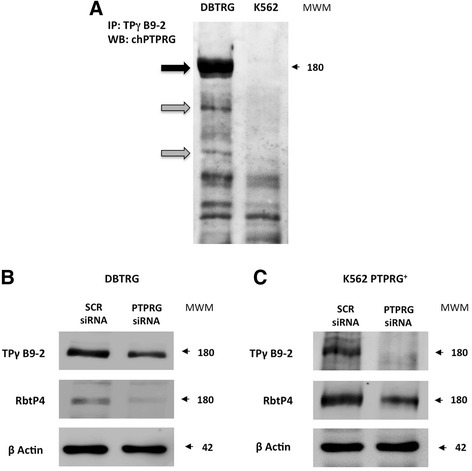
TPγ B9-2 specifically recognizes PTPRG. Downregulation of PTPRG by siRNA demonstrates the specificity of TPγ B9-2 monoclonal antibody for the antigen. Immunoblotting was performed with the indicated antibodies after transfection with a specific PTPRG siRNA (siRNA) and with a negative control siRNA (scrambled: SCR). Cell lines were DBTRG and K562, and antibodies were Mab TPγ B9-2 or the reference rabbit polyclonal antibody RbtP4 and chicken (ch) anti PTPRG [9, 10]. **a** Immunoprecipitation of PTPRG by TPγ B9-2 monoclonal antibody. K562 and DBTRG cell lines, respectively negative and positive for PTPRG mRNA expression, were subjected to immunoblotting analysis with chPTPRG antibody after immunoprecipitation with TPγ B9-2 antibody. *Left side*: *Black arrow*, full-length PTPRG; *gray arrows* indicate putative processed forms. No signal was detectable using an irrelevant antibody for IP (data not shown). **b** Western blotting with mab TPγ B9-2 or the reference rabbit polyclonal antibody RbtP4 in PTPRG expressing DBTRG cell line treated with scrambled (SCR) or PTPRG-specific siRNA. Both antibodies detect the downregulation of PTPRG. Anti-β-actin was used as a loading control. **c** Western blotting with mab TPγ B9-2 or the reference rabbit polyclonal antibody RbtP4 in PTPRG silenced K562 cell lines overexpressing PTPRG (K562 PTPRG^+^). Downregulation of the 180 kDa band is apparent in silenced cells using both antibodies. Differences in signal intensities are due to the combined effect of individual affinities of primary antibodies toward the native or cDNA-transfected antigens and secondary antibodies toward the murine or rabbit Igs

We have developed a monoclonal antibody using as antigen the whole ECD of PTPRG purified from cDNA-transfected HEK293F cells. The antigen is a glycoprotein, the epitopes of which can greatly influence the capability of the resulting antibodies to recognize the native antigen. We previously evaluated the capability of affinity-purified TPγ B9-2 to recognize the full-length protein by immunoprecipitation in the K562 cell line transfected with PTPRG cDNA [8]. Glioblastoma expresses one of the highest levels of PTPRG [19]. Here, by western blotting, we confirmed specificity of TPγ B9-2 in a glioblastoma cell line DBTRG overexpressing endogenous PTPRG (Fig. 1a).

We next specifically downregulated PTPRG using siRNA in K562 expressing PTPRG cDNA under the control of the CMV promoter [8] (Fig. 1b) and in DBTRG (Fig. 1 c–d) PTPRG expressing cells.

Of note in DBTRG cells, where PTPRG is endogenously expressed, both monoclonal TPγ B9-2 and chPTPRG recognized two isoforms of PTPRG (Fig. 1a), the putative full-length protein of approximately ≈180 kDa and minor ≈150 and ≈100 kDa putative processing products that were reported to come up when the protein is overexpressed [9].

### Immunohistochemistry analysis

**Fig. 2:**
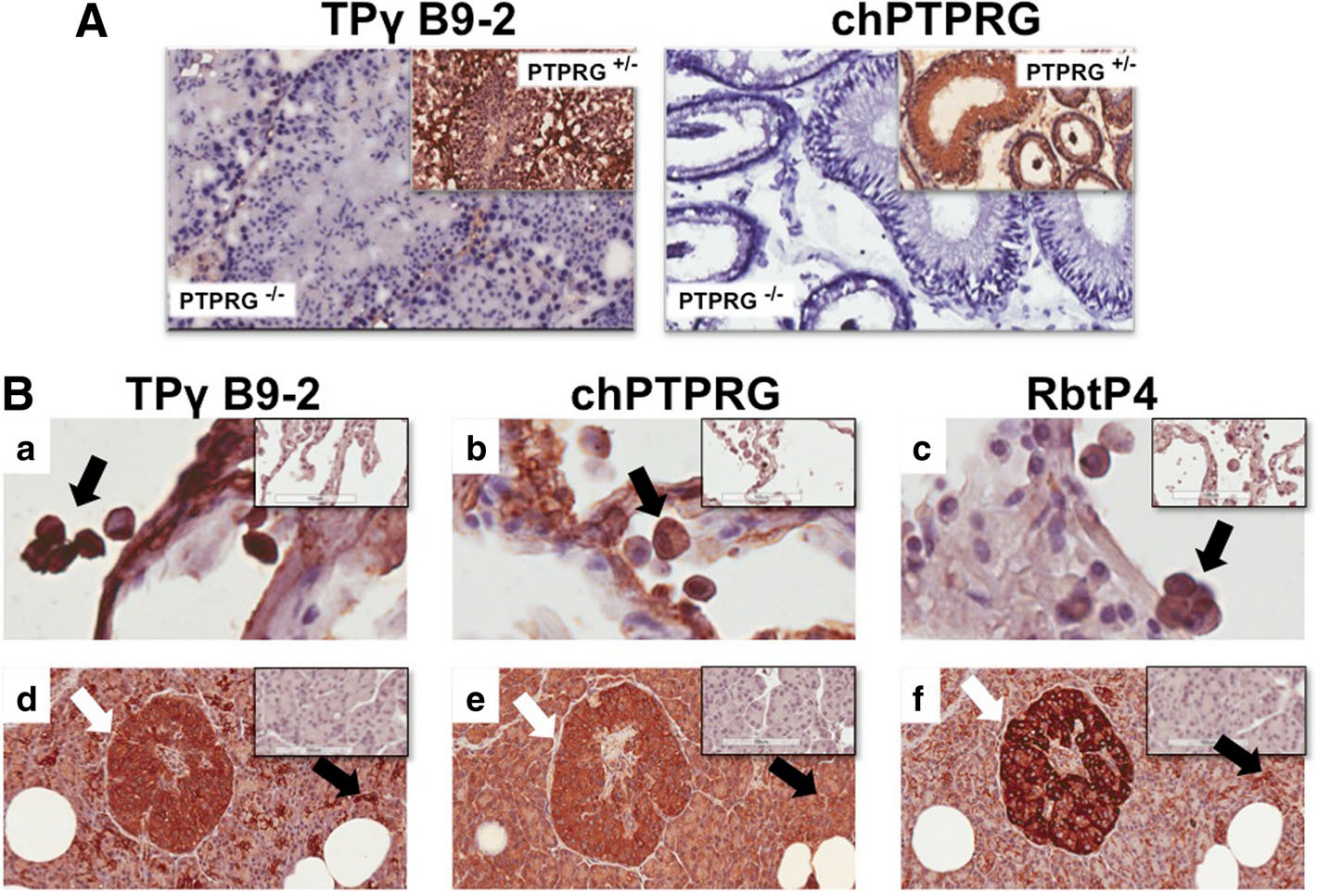
In situ analysis of PTPRG expression. **a** Immunohistochemistry using monoclonal TPγ B9-2 and chPTPRG antibodies on cryostatic sections of mouse testis. In the heterozygous PTPRG (+/−) mice, shown in the *insets*, the two antibodies recognize the same structures while in Ptprg-null (KO) (−/−) mice, shown as the main figure, no staining was detectable. **b** Comparison between TPγ B9-2, chPTPRG, and RbtP4 in normal human lung (*a*–*c*) and pancreas (*d*–*f*). Formalin-fixed, paraffin-embedded sections were stained with anti-PTPRG antibodies indicated. All the antibodies recognize lung alveolar macrophages (*a*–*c*, *arrows*). In pancreas (*d*–*f*) TPγ B9-2 preferentially stains Islets of Langerhans (*white arrows*) and centroacinar regions (*black arrows*), while chPTPRG stains the tissue more diffusely, including the exocrine glands. Matched irrelevant antibodies controls (murine IgG_1_, IgY, and rabbit IgG) are shown in the *insets*

Human and murine ECDs of PTPRG (accession numbers AAA60224.1 and Q05909, respectively) share 91.2% identity and 95.0% similarity at the amino acid level as calculated using the Needleman-Wunsch global alignment algorithm. Therefore, cross-reactivity of the antibody between these species was predictable. To evaluate this possibility and unequivocally establish the capability of the antibody to specifically recognize the antigen, we tested a tissue sample of Ptprg-null (KO) and matched heterozygous mice [20] as negative and positive controls, respectively. TPγ B9-2 recognized PTPRG only in samples obtained from the testis of control heterozygous mice (Fig. 2a, left). The specificity of the signal was further confirmed by the positive staining obtained with a previously validated antibody developed in chicken (named chPTPRG) against the 20 amino acid peptide CZNEDEKEKTFTKDSDKDLK, a highly conserved sequence among vertebrates with a 100% homology among human, mouse, and rat (Fig. 2a, right) [19]. The TPγ B9-2 antibody also recognized human formalin-fixed, paraffin-embedded tissues, as shown in Fig. 2b (a, d). This experiment further demonstrated the specificity of the staining as well as the identity of the histological structures highlighted by the monoclonal antibody as we reacted samples from the normal lung and pancreas with two additional polyclonal antibodies we previously developed in chicken and rabbit against extracellular epitopes of the antigen (named chPTPRG and RbtP4), respectively (Fig. 2b (b, e and c, f)) [19, 21]. All the antibodies showed an overlapping pattern of staining, with no detectable signal when matched with irrelevant controls used in consecutive sections (insets). All the antibodies recognized lung macrophages (black arrows; Fig. 2b (a–c)) and endocrine cells within the pancreas (Islets of Langerhans, white arrows), along with centroacinar cells (black arrow; Fig. 2b (d–f)), as previously described [21]. The chPTPRG stained exocrine cells more diffusely [19, 22]. As PTPRG is known to be expressed in various isoforms, this result might be explained by the presence of a differentially spliced/processed form of the phosphatase within pancreatic tissue.

**Fig. 3:**
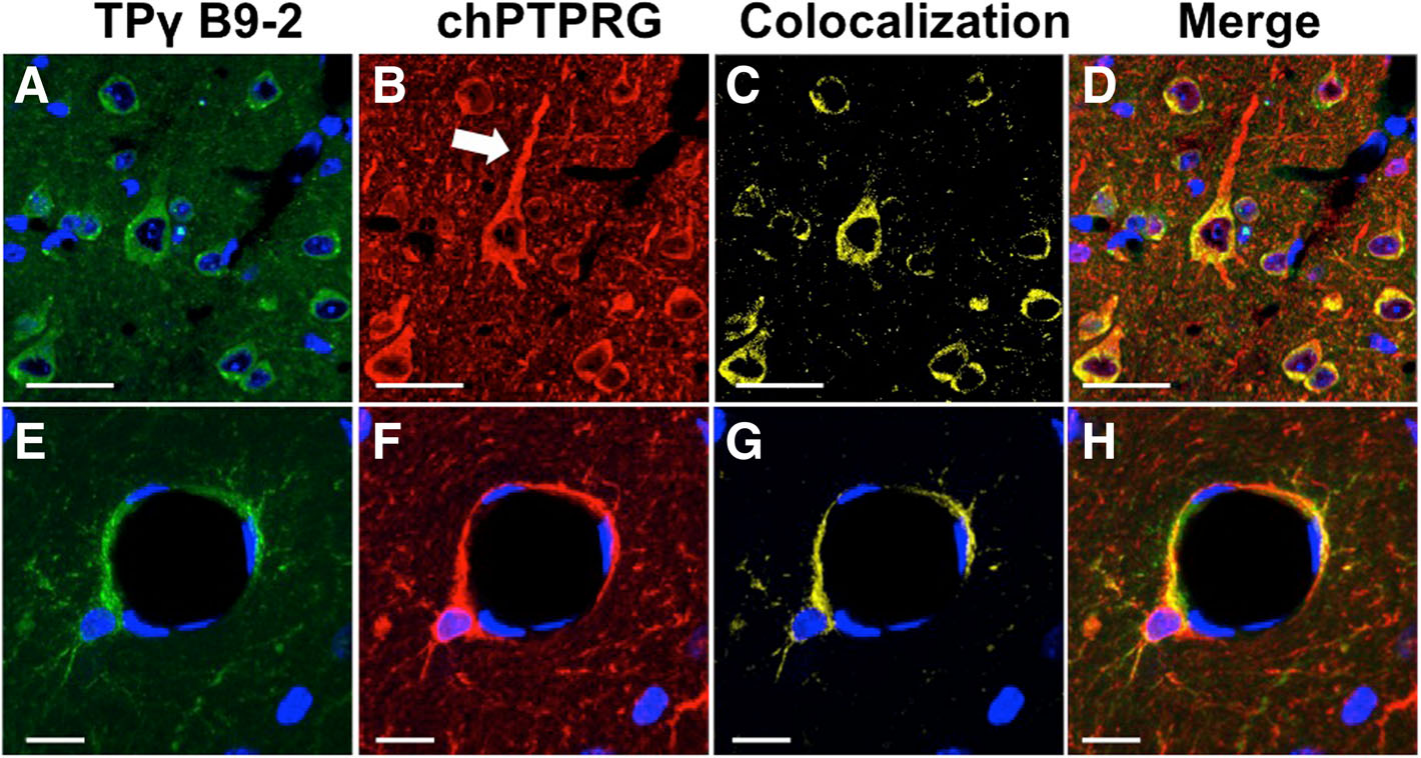
Co-localization of different anti-PTPRG antibodies on the same histo-cytologic structures: murine cerebral cortex (**a**–**d**) and vessel sections (**e**–**h**) were analyzed using TPγ B9-2 and chPTPRG antibodies. *White arrow*, a dendrite staining positive for chPTPRG and negative with TPγ B9-2 (**b**). A slightly different subcellular distribution is also observed in small vessels as shown by overlapping of both stains (in colocalization and merge mode, **c**, **d**, **g**, **h**). In colocalization mode, *yellow regions* indicate only the overlapping epitopes. Merge mode shows signals from the individual antibodies highlighting the capability of chPTPRG (in *red*) to identify dendrites that are not recognized by TPγ B9-2 (in *green*). Slight differences in staining are present also in vessel sections. These results confirm the presence of different Ptprg isoforms recognized by the antibodies and localized in different cellular structures. DAPI (*blue*) stains nuclei. *Scale bars*, 30 μm in **a**–**d**, 10 μm in **e**–**h**

Once we demonstrated the specificity of the reagents employed, we then assessed whether the monoclonal and chicken antibodies shared the same epitope by treating the same murine brain section with both antibodies. One was directly labeled with Alexa fluor 488 (TPγ B9-2), while the second (chPTPRG) was visualized using a NorthernLights 637 Fluorochrome-labeled secondary antibody. The results suggested that the epitopes recognized are different as minor differences among subcellular structures were observed, despite an almost complete overlapping of the histo-cytologic details recognized by both antibodies as shown by Fig. 3c, g (co-localization of both antibodies) and Fig. 3d, h (merging of both antibodies). Indeed, while both TPγ B9-2 (Fig. 3a) and chPTPRG (Fig. 3b) antibodies correctly recognized the neuronal soma in murine brain, only the latter recognized the dendrites (white arrow), again suggesting that the antibodies can recognize different isoforms of the phosphatase.

### Flow cytometry analysis

**Fig. 4:**
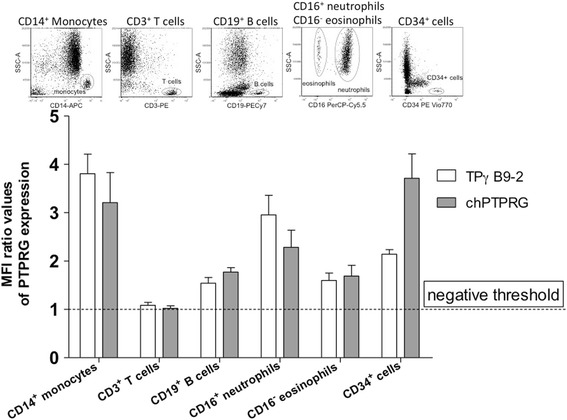
Flow cytometric analysis of healthy human donor peripheral blood samples using two anti-PTPRG antibodies. *Dot plots* show gating strategy for each cell subset analyzed in histograms. *N* = 34 monocytes, *N* = 20 T cells, *N* = 26 B cells, *N* = 16 neutrophils, *N* = 10 eosinophils, and *N* = 18 CD34+ cells. PTPRG expression in peripheral blood circulating monocytes (CD45^+^; CD14^+^), T cells (CD45^+^; CD3^+^), B cells (CD45^+^; CD19^+^), PMN neutrophils (CD45^+^; CD16^+^), PMN eosinophils (CD45^+^; CD16^−^), CD34^+^ progenitor cells (CD45^low^; CD34^+^), using the reference antibody chPTPRG and TPγ B9-2. Data are expressed as mean fluorescence intensity (MFI) with appropriate isotype controls (preimmune chicken IgY and irrelevant murine IgG_1_) as references

**Fig. 5:**
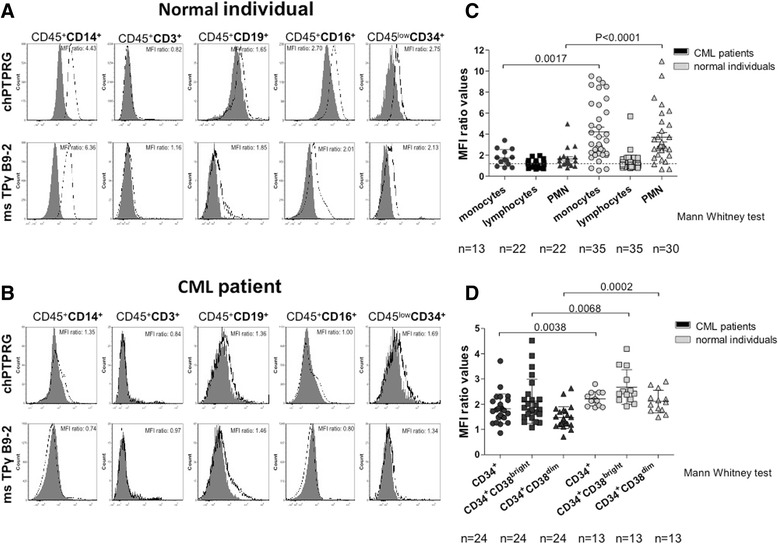
Flow cytometric analysis using TPγ B9-2 antibody of normal individuals and CML patients. Representative flow cytometric analysis of a normal individual (**a**) and a CML patient (**b**) performed with both chicken and Mab for comparison. **c** Comparison made using mean fluorescence intensities (MFI) values obtained with TPγ B9-2. **d** PTPRG expression in CD34^+^, CD34^+^CD38^bright^, CD34^+^CD38^dim^ subpopulations from 13 G-CSF mobilized normal individuals and 24 CML patients. MFI values obtained calculating the ratio between the signal derived from TPγ B9-2 and irrelevant murine IgG_1_, respectively (*p* values for statistic derive from Mann-Whitney test)

**Fig. 6:**
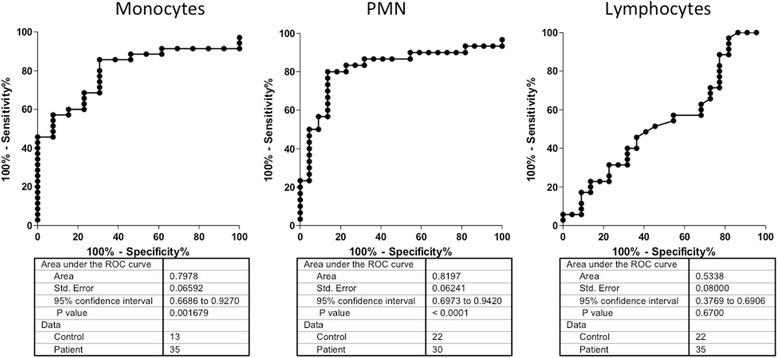
Evaluation of sensitivity and specificity of the test. A receiver operating characteristic curve (ROC curve) was built for normal individuals vs CML patients. Data obtained with TPγ B9-2 derived from patients shown in Fig. 5. A statistically significant capability to discriminate between CML and healthy subject is achieved when monocytes and PMN are evaluated. No/negligible expression of PTPRG in lymphocytes renders these cells unsuitable for the purpose

The TPγ B9-2 antibody was originally selected for its capability to specifically recognize the antigen by flow cytometry, as no commercial antibody had been reported for this important application. We evaluated TPγ B9-2 capability to detect the native form of PTPRG by performing a side-by-side comparison of the capability of chPTPRG and TPγ B9-2 antibodies to recognize the same major leukocyte subpopulations (gating strategy displayed in upper Fig. 4) in peripheral blood of normal donors (lower Fig. 4). In a population of CD45^+^ cells, we observed that both antibodies readily recognized CD14^+^ monocytes, CD16^+^ neutrophils, CD19^+^ B cells, CD16^−^ eosinophils, and CD34^+^ progenitor cells. We observed lower/undetectable levels of PTPRG expression in CD3^+^ T cells (Fig. 4). These results are in line with those previously obtained using the chicken antibody [10]. In Fig. 5a–c, we have compared side-by-side the mean fluorescence intensity (MFI) measured in the same cell populations of a series of normal individuals and CML patients (see Additional file 1: Table S1 for details) using TPγ B9-2 and chPTPRG antibodies and confirmed PTPRG downregulation in monocytes (*p* = 0.0017) and PMN (*p* < 0.0001) in CML patients in comparison with healthy controls (Fig. 5a–c). The downregulation of PTPRG was shown across both mature and primitive CD34 subsets (*p* = 0.0068; CD34^+^CD38^bright^ and *p* = 0.0002; CD38^dim^ cells (Fig. 5d)). We then built a receiver operating characteristic curve (ROC curve) for normal individuals and CML patients. A statistically significant capability to discriminate between CML and healthy subject is achieved when monocytes and PMN are evaluated. No/negligible expression of PTPRG in lymphocytes renders these cells unsuitable for the purpose (Fig. 6).

### Downregulation of PTPRG in CML and recovery of normal PTPRG^+^ cells during the remission phase of the disease

**Fig. 7:**
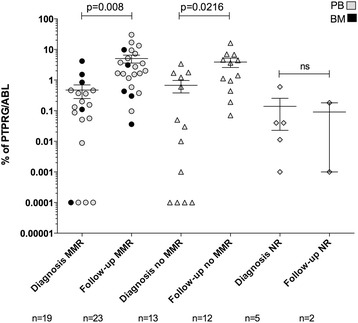
PTPRG mRNA expression in paired samples of patients treated with TKI. RQ-PCR analysis of PTPRG expression in peripheral blood (gray symbols) and bone marrow (black symbols) calculated as % vs ABL1 mRNA. Major molecular response (MMR) is associated with recovery of PTPRG expression. Only the few non-responder patients failed to recover PTPRG expression

**Fig. 8:**
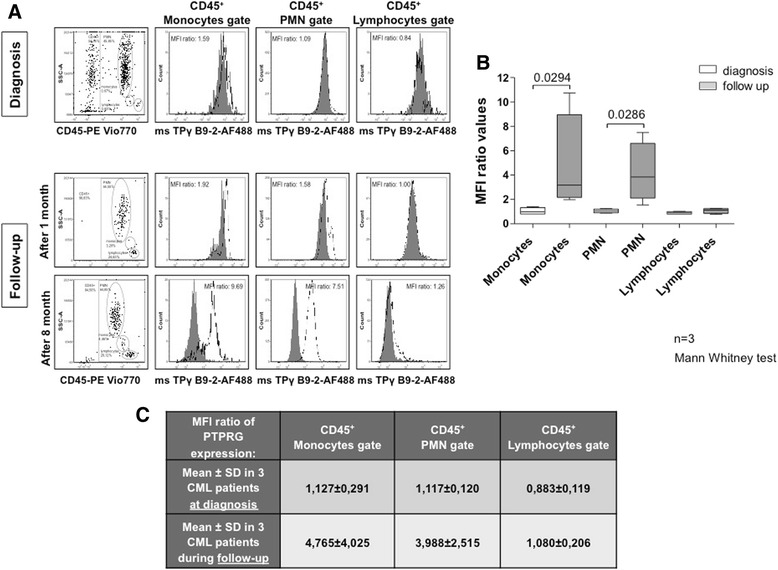
PTPRG expression in various leukocyte populations of CML patients at diagnosis and after treatment. **a** Representative flow cytometric analysis of PTPRG protein at diagnosis and at follow-up following successful TKI-based treatment. At diagnosis, monocytes and PMN downregulate PTPRG expression. After an average of 6 months of treatment, the levels of phosphatase expression recover and become similar to healthy donors. **b** *Box-Whisker diagram* displays significant differences between PTPRG expression in monocytes and PMN populations in three different CML patients at diagnosis and after treatment (statistical analyses are indicated on graphs). **c** The table summarizes the mean fluorescence intensity (MFI) values related to PTPRG expression shown in panel **b**

Finally, we asked whether we could confirm and extend our previous observation regarding the loss of PTPRG in CML [8]. We performed a retrospective analysis of a group of 39 CML patients (described in Additional file 1: Table S2) evaluated at diagnosis and after the indicated treatment by measuring PTPRG messenger RNA (mRNA) levels by RQ-PCR. Among these, we could perform analysis for 33 individuals, whose data are summarized in Fig. 7. We considered whether the failure to achieve a major molecular response (MMR) was associated with decreased/absent recovery of PTPRG. For patients achieving MMR (*p* = 0.008) and for those with intermediate responses (*p* = 0.0216; no MMR), PTPRG levels showed a significant increase when baseline was compared to follow-up after treatment. However, the few patients who proved to be non-responsive to treatment (NR) displayed the lowest PTPRG mRNA levels that did not increase following treatment (Fig. 7). Finally, we analyzed fresh peripheral blood samples from three patients at diagnosis and following successful TKI-based therapy. In these cases, downregulation of PTPRG protein expression was confirmed at diagnosis, while its expression recovered in tandem with the return of Philadelphia-negative hematopoiesis and achievement of MMR in response to effective TKI treatment (Fig. 8a–c).

## Discussion

*PTPRG* is emerging as a key tumor suppressor gene in many types of neoplasia [5, 7, 23–25]. More recently, an oncosuppressive role has been described in selected hematologic malignancies [8, 26], suggesting that the availability of an antibody suitable for flow cytometric applications would represent a valuable tool with potential clinical applications. In this study, we present the characterization of the first monoclonal antibody raised against the ECD of human PTPRG, expressed and purified from the HEK293F human cell line, which was found suitable for different immunoassay applications, including flow cytometry. This monoclonal antibody (named TPγ B9-2) appears capable of recognizing murine and human PTPRG by immunofluorescence, immunohistochemistry, and flow cytometry and its reactivity overlaps almost entirely with the tissue structures recognized by another validated polyclonal antibody, chPTPRG, raised against a peptide present in the ECD of PTPRG [10].

Of interest is the observation that TPγ B9-2 displays a slightly different subcellular reactivity, although recognizing overlapping structures in human and murine tissues, suggesting its capability to recognize a distinct epitope associated with a specific isoform. The presence of PTPRG isoforms was described in previous work carried out in rats [22], humans, and mice [19, 21]. This observation might also help to explain slight differences in reactivity found in normal and neoplastic samples (in both hematological and solid tissue samples) that will need to be further evaluated in future studies. Importantly, both antibodies confirmed that PTPRG is downregulated in the leukocytes of patients affected by CML, including CD34^+^CD38^bright^ and CD34^+^CD38^dim^ hematopoietic precursors. This observation implies that its loss occurs at the stage of early hematopoietic precursors. PTPRG is a tumor suppressor gene capable of interfering with BCR-ABL1 signaling by decreasing BCR-ABL1-dependent tyrosine phosphorylation in CML cells [8]. Its downregulation in all CD34^+^ fractions of CML patients along with the demonstration that its forced up- and downregulation in CML cells reduces and increases, respectively, clonogenic capacity [8], suggesting a key role in the maintenance of the disease. The role of PTPRG in controlling hematopoietic cell survival/differentiation is further supported by an early study [3], while the most recent evidence linking PTPRG to the pathogenesis of leukemia would be in keeping with inhibition of PTPRG expression by epigenetic regulation consisting in methylation of selected promoter regions and the fact that its downregulation cooperates with RAS gene activation in childhood acute lymphoblastic leukemia (ALL) [26]. This last study adds another hematological malignancy to the number of diseases where deregulated PTPRG is disease associated. Of additional interest is the new observation that TPγ B9-2 antibody is capable of detecting PTPRG expression in non-CML hematopoietic cells re-emerging in the same patient after effective therapy, thus confirming that normal hematopoiesis does require an appropriate level of PTPRG expression, and the latter is specifically downregulated in the diseased cells. Under these carefully controlled conditions (same individuals-same antigen) an antibody found specific for PTPRG binds to the cell surface after TKI therapy. This suggests that the antigen is now expressed as happens for normal individuals. Very few examples of cell surface biomarkers specifically expressed in CML are available in the literature; however, the ones described are expressed on the cell surface and not downregulated or lost, like PTPRG, in CML cells. Among these, RNA expression analysis identified an association between low CD7 expression along with high expression of proteinase 3 or elastase with longer survival in CML [27]. An association was described between hypomethylation of CD7 promoter region and its increased expression [11] The orphan receptor tyrosine kinase ROR1 was shown to be expressed in follicular lymphoma. Also, chronic lymphocytic leukemia, hairy cell leukemia, and CML had significantly higher numbers of ROR1^+^ cells [13]. Sperm-associated antigen 9 (SPAG9) mRNA and protein were found to be expressed in CML patients (88%) and have been associated with humoral response against this antigen in 90% of the cases, suggesting it as a potential biomarker and/or immunotherapeutic target [12]. No specific functional roles in CML have been associated with this gene product. A proteomic survey of cell surface proteins in leukemia cell lines identified 25 proteins, including CD43 and CD98, which appeared to be differentially expressed in CML cell lines, with CD43 expressed uniquely in K562 cells [28]. More recently, a series of genes were identified by transcriptome analysis [14]. Both IL1RAP and CD26 can be regarded as promising candidates as CML-specific antigens in the CD34^+^CD38^−^ CML LSC population [15–17]. Within this context, PTPRG has unique features as it is specifically downregulated in CML and has been demonstrated to have a functional role being capable of binding and dephosphorylating the driving oncoprotein BCR-ABL1 and consequently reducing total and specific phosphotyrosine levels as well as clonogenic capacity in various CML cells [8]. Of interest is the recent observation that JAK2 kinase, a key signaling molecule involved in BCR-ABL1-driven pathways [29], represents a bona fide PTPRG substrate [30].

## Conclusions

PTPRG represents a remarkable example of a surface protein biomarker with a specific oncosuppressive mechanism intersecting with BCR-ABL1 and key elements in its signaling pathway, making it an attractive target for the development of dedicated reagents, such as specific agonists [30]. Indeed, the development of a specific monoclonal antibody is expected to help unravel the complex biological role of this phosphatase. The evaluation of larger series of leukemia samples (myeloproliferative diseases, CML, and ALL) for PTPRG expression by flow cytometry, in addition to in situ techniques, as well as the detailed definition of its signaling pathway, may reveal additional details regarding CML biology. This in turn might pave the way for the evaluation of its suitability for clinical application by supporting diagnosis and follow-up of these disorders and as a potential therapeutic target.

## Additional file

Additional file 1: Supplementary material and methods.

## Abbreviations

chPTPRG: Anti-PTPRG antibody developed in chicken
CML: Chronic myeloid leukemia
ECD: Extracellular domain
MMR: Major molecular response
PTPs: Protein tyrosine phosphatases
TKI: Tyrosine kinase inhibitors
PTPRG: Tyrosine phosphatase receptor gamma
