# Supplementary material for: A new monoclonal antibody detects downregulation of protein tyrosine phosphatase receptor type γ in chronic myeloid leukemia patients
Source: J Hematol Oncol. 2017 Jun 21;10:129. doi: 10.1186/s13045-017-0494-z (PMC5479035; doi:10.1186/s13045-017-0494-z)
Supplement: Supplementary file 1 — Supplementary material and methods. [file 13045_2017_494_MOESM1_ESM.docx]

**Supplementary Material and Methods**

**Cell Culture and transfection**

HEK293F cells were transfected and grown in RPMI 1640 containing 10% heat-inactivated Fetal Calf Serum (FCS, Lonza, Basel, Switzerland), 4 mM glutamine and 0.5 mg/mL of hygromycin (Thermo Fisher, Milan, Italy) to select transduced cells or cultured in CD293^®^ serum free medium (Thermo Fisher, Milan, Italy) with 50 µM β-mercaptoethanol and 0.5 mg/ml of hygromycin. K562 CML cell lines were transfected with empty vector (mock) and PTPRG full length cDNA (γ1) as described (8) and cultured in RPMI 1640 supplemented with 10% heat-inactivated FBS (Lonza), 0.50 mg/mL G418 (Thermo Fisher, Milan, Italy) and 2 mM Ultraglutamine (Lonza). DBTRG, glioblastoma multiforme cell line and LAMA-84, CML cell line, were cultured in the same growth medium without G418. All the cells were grown at 37°C, in 5% CO_2_.

**Extraction of proteins**

Cells at concentration of approximately 20x10^6^/mL were solubilized in lysis buffer (LB) containing 50 mM Tris (pH 7.4), 0.5% Nonidet P-40, 150 mM NaCl, 10% glycerol and Complete EDTA-free Protease Inhibitor Cocktail Tablets (Roche, Milan, Italy), incubated for 15 minutes in a rotating wheel at 4°C and centrifuged for 10 minutes at 4°C at 16000×g in a Biofuge Heraeus (DJB Labcare Ltd. UK). Total protein content was assessed using Bradford assay (Sigma, Missouri, USA).

**RNA isolation, reverse transcription, real-time qRT–PCR**

Blood peripheral blood samples, taken during diagnostic and follow up routines, were collected into BD Vacutainer Plastic K3EDTA tubes; erythrocytes were lysed using lysis buffer (165 mM NH_4_Cl, 10 mM KHCO_3_, 0.2 mM EDTA, pH 8.0) and white cells separated by centrifugation at 270×g. The collected cells were resuspended in TRIzol reagent (Thermo Fisher Italia, Italy) and stored at -80°C. Total RNA was isolated following the manufacturer’s instructions.

For cDNA synthesis, 1µg of total RNA was reverse-transcribed in a 25µL reaction volume, with random hexamer primers, using the RT Kit plus (Elitech Group, Italy) following the manufacturer’s protocol. Briefly, the reaction was preincubated for 10 min at 20°C to allow annealing of random hexamers followed by reverse transcription at 42°C for 45 min and denaturation at 99°C for 3 min to inactivate the reverse transcriptase.

RQ–PCR for PTPRG, BCR-ABL1 and ABL1 was performed using a 7500 Real Time system (Thermo Fisher Italia, Italy) using predesigned TaqMan quantitative RT-PCR assays for PTPRG (intracellular domain) and ABL1 genes, while quantification of BCR-ABL1 gene fusion transcripts was done according to the European Leukemia Net guidelines (28).

PCR was performed in a 20 μL reaction volume containing 2,5 μL cDNA template, 10 µl of 2X universal master mix and 1 µL of the assay. In order to generate standards, the PTPRG full length cDNA was cloned in EcoR1 site of PCR3.1 plasmid(8). The PTPRG plasmid DNA was measured by spectrometry to assess the copy number. Standard curves were then prepared using ten-fold serial dilutions, ranging from 2x10^5^ to 2x10^2^ copies of plasmid pCR 3.1 PTPRG and stored at ­-20°C until use to prevent degradation. Plasmids for BCR-ABL1 and ABL1 were commercially available from Elitech Group, Italy.

PCR program started with an incubation at 94°C for 10 min to activate the Taq DNA polymerase, followed by 50 cycles of amplification, each involving a denaturation step at 94°C for 15 sec and an annealing–extension step at 60°C for 1 min.

All samples were analyzed in duplicate. The results were expressed as the PTPRG or ABL1 copies and normalized levels were calculated as the PTPRG/ABL1 ratio. The expression of ABL1 control gene showed a median Ct of 24.91 (range 23.67-27.01). Samples for which the Ct of ABL1 was >29 cycles were considered invalid and therefore excluded.

**Immunoprecipitation**

Protein G-Sepharose beads (Sigma, 30 µL of for each sample) were washed once with cold TBS and once with cold protein LB and incubated with 3 μg of monoclonal antibody TPγ B9-2 in 250 µL of LB for 1 hour at 4°C with gentle rocking. 500 μg of total protein content resuspended in LB with Complete EDTA-free Protease Inhibitor Cocktail tablets were added to the affinity matrix and incubated for 3 hours at 4°C with gentle rocking. Antibody-conjugated beads were washed twice with cold LB and once with cold TBS, resuspended in Sample Buffer, incubated at 95°C for 10 minutes and subjected to SDS-PAGE.

**Western blot analysis**

Total protein extract (30 µg) were mixed to 0.25 volumes of Sample Buffer (160 mM Tris, 20% glycerol, 5% β-mercaptoethanol, 4% SDS, 0.01% bromophenol blue) and loaded on a 6% polyacrylamide, 0.1% SDS gel. Gels were run for 2 hours in a Mini Protean 3 Apparatus (Biorad, Milan, Italy) with 0.3% Tris, 1.45% Glycine, 0.1% SDS running buffer, pH 8.9.

After SDS-PAGE, gels were electro-blotted on polyvinylidene difluoride membrane (Sigma Aldrich, Milan, Italy) for 1 hour and 15 minutes at 100V, 135 mA (2.5 mA/cm^2^) in a Mini Trans-Blot Electrophoretic Transfer Cell (Biorad); For Rbt anti-P4 and TPγ B9-2 antibodies, membranes were saturated by incubation for 1 hour in TBS-0.05% Tween^®^ 20/BSA 5%. Then membranes were incubated overnight with Rbt anti-P4 and TPγ B9-2 (1μg/mL) and β-Actin (Sigma Aldrich,) (0.1 µg/mL), diluted in TBS-0.05% Tween^®^ 20/BSA 1%, then washed three times with TBS-0.05% Tween^®^20 and incubated for 1 hour with appropriate HRP-conjugated secondary antibodies, ECL Rbt IgG, HRP-linked F(ab)_2_ fragment from donkey (GE Healthcare Life Sciences, Buckinghamshire, UK) and Anti-Mouse IgG (Fc specific)–Biotin antibody produced in goat (Sigma Aldrich), diluted in TBS-0.05% Tween^®^ 20/BSA 1%. After three further washes, membranes were assayed with ECL (Millipore, Billerica, MA, USA).

**Small interfering RNA** (**siRNA) transfection**

siRNA targeting PTPRG (siPTPRG, n° s-11550) and negative control (scrambled) were purchased from Applied Biosystems^®^ Thermo Fisher. Cells, at concentration of 1x10^5^/mL, were transfected with 30 nM (final concentration) of siRNAs using siPORT™ NeoFX™ Transfection Agent (Applied Biosystems^®^ Thermo Fisher), according to the manufacturer's instructions. Cells were cultured for 72 hours, washed twice with cold TBS and lysed.

**Flow Cytometry**

Erythrocytes were lysed adding 1 mL of red blood cell LB ((165 mM NH_4_Cl, 10 mM KHCO_3_, 0.2 mM EDTA, pH 8.0)) to 100 µL of peripheral blood/bone marrow. Cells were washed twice with PBS 1X and then incubated for 20 minutes with the following antibodies: anti-CD45-Vioblue (130-092-880 Miltenyi Biotec), anti-CD34 PE-Cy7 (25-0349-42 eBioscience), anti-CD38-APC (130-092-261 Miltenyi Biotec), anti-CD14 PE-Cy7 (325618 BioLegend), anti-CD19 PE-Cy7 (302216 BioLegend), anti-CD16 PE (130-091-245 Miltenyi Biotec). After washing with PBS 1X, cells were incubated for 15 minutes with 100 µL of a blocking solution (human IgG 10μg/mL in PBS 1X) followed by 1 µg of mouse IgG_1_ K Isotype Control Alexa Fluor^®^ 488 (53-4714-42 eBioscience, San Diego, CA), mouse anti-human PTPRG TPγ B9-2 antibody-AF488, pre-immune chicken IgY and chicken anti-human PTPRG. After an incubation of 30 minutes in the dark and two washes with PBS 1X, the secondary Goat anti-Chicken IgY (H+L) Alexa Fluor® 488 conjugate (A11039 Thermo Fisher) 1:200 was added for 30 minutes, followed by a final wash in PBS 1X. Flow cytometry was performed on a MACSQuant^®^ Analyzer (Miltenyi Biotec). Analysis of data was performed with FCS Express 4 Plus Research Edition (De Novo Software).

**Immunohistochemistry**

Heterozygous and PTPRG deficient mice (129SveV, provided by Dr. S. Harroch from the Institut Pasteur of Paris, France) were transcardially perfused with paraformaldehyde 4 % in saline buffer . Samples of testis were post-fixed with 4% PFA/20% sucrose and then cut in 7-mm thick sections as described (17). Endogenous peroxidase and non-specific sites were blocked by incubation with 3% H_2_O_2_ and 1% BSA, respectively, for 30 min. at room temperature.

Paraffin-embedded human tissues were cut into 4-μm thick sections and placed on polylysinated slides. Sections were dewaxed, rehydrated with graded alcohol and antigen retrieval was performed in 10 mM citrate buffer, pH 6.0 heated in a microwave at 360 W for 20 minutes.

Endogenous peroxidase and non-specific sites were blocked by incubation with 3% H_2_O_2_ and PBS/ Tween^®^20 0.05%/BSA 1%/NaN_3_ 4 mM, respectively, for 15 minutes at room temperature.

Sections were then incubated with the following primary antibodies: chicken anti-PTPRG (6.25 μg/mL) or mouse anti-PTPRG TPγ B9-2 (5 μg/mL) or Rbt anti- P4 (5 μg/mL) for 90 minutes at room temperature in PBS/ Tween^®^20 0.05%/BSA 1%/NaN_3_ 4 mM.

Then sections were washed three times with PBS 0.2% Tween^®^20and incubated for 40 minutes at room temperature with the appropriate secondary antibodies. These included: rabbit anti-chicken IgY (IgG) (whole molecule)-HRP (A9046 Sigma-Aldrich, 1:1000), goat anti-mouse conjugated to peroxidase-labelled dextran polymer (K 4001, DakoEnVision+ Peroxidase, Mouse Ready-to-use) and goat anti-rabbit Histofine^®^ Simple Stain™ MAX PO (414142F, Nichirei Corporation, Tokyo, Japan).

After three washes with PBS 0.2% Tween^®^ 20, the immunoreaction was visualized using 3, 3’-diaminobenzidine (DAB) staining (K3648, Dako). Finally, the sections were counterstained with hematoxylin and dehydrated. For negative controls, the primary antibodies were replaced with the appropriate isotypes.

**Immunofluorescence**

C57/BL6J mice purchased from Harlan were maintained under standard environmental conditions (temperature, humidity, 12 h/12 h light/dark cycle, with water and food ad libitum) under veterinarian assistance. Animal handling and surgery were performed under the Animal Care and Use Committee of the University of Verona (CIRSAL), and authorization by the Italian Ministry of Health, in strict adherence to the European Communities Council (86/609/EEC) directives, minimizing the number of animals used and avoiding their suffering.

Under deep anesthesia (tribromoethanol, intraperitoneal dose of 0.5 g/kg body weight) mice were transcardially perfused with paraformaldehyde 4% in saline buffer. The brain was excised, post-fixed and cryoprotected before freezing. Brain slices (35 µm thick) were obtained and stained with antibodies.

Free-floating cryosections were stained with different combinations of antibodies. Sections were permeabilized with the following solution: 2 % bovine serum albumin and 0.3% Triton X-100 in PBS, pH 7.4 for 30 minutes. Then sections were incubated overnight at 4°C with either Alexa Fluor488-conjugated TPγ B9-2 or with chPTPRG antibody followed by goat anti-chicken IgY (NorthernLights 637 Fluorochrome-labeled Antibody NL637 from R&D, Minneapolis, MN, USA) for 1 hour at room temperature. Nuclei were stained with DAPI (Sigma). After mounting with an anti-fading solution, slices were studied by fluorescence microscopy (DM6000B, Leica Microsystem) and confocal microscopy (TCS-SP5, Leica Microsystem).

**Statistics**

SigmaStat software version 3.0 (SPSS Inc., Chicago, U.S.A.) and Prism software version 5.0 (La Jolla, CA 92037 U.S.A.) were used for statistical calculations. According to the distribution of results, parametric and/or nonparametric statistics were applied to test hypotheses on the means and/or the medians. The α value was set to 0.05 and the level of significant difference to P < 0.05.

Table S1: Clinical features of CML patients for PTPRG protein expression analysis

| **CML#** | **Age** | **Gender** | **Basophils %** | **WBC (10^6^/L)** | **PB blasts %** | **BM blasts %** | **SOKAL risk** | **EUTOS risk** |
| --- | --- | --- | --- | --- | --- | --- | --- | --- |
| 1 | 28 | M | 0,0 | 123,40 | 4,0 | < 5 | Intermediate | low |
| 2 | 65 | M | 5,0 | 29,50 | 2,0 | n.a. | Intermediate | low |
| 3 | 62 | M | 17,0 | 36,29 | 1,0 | n.a. | Intermediate | high |
| 4 | 58 | F | 0,0 | 29,05 | 1,0 | 2,0 | Intermediate | low |
| 5 | 37 | M | 1,0 | 69,60 | 1,0 | < 5 | Intermediate | low |
| 6 | 45 | F | 11,0 | 26,62 | 0,0 | 1,0 | High | low |
| 7 | 61 | F | 9,0 | 41,97 | 1,0 | 0,0 | Intermediate | low |
| 8 | 32 | M | 4,0 | 360,50 | 1,0 | 4,0 | Intermediate | high |
| 9 | 53 | F | 2,0 | 46,50 | 0,0 | 1,0 | Low | low |
| 10 | 50 | F | 3,0 | 83,40 | 0,0 | 1,0 | Low | low |
| 11 | 74 | M | 3,0 | 43,06 | 0,0 | 1,0 | Intermediate | low |
| 12 | 75 | F | 5,0 | 186,40 | 2,0 | 2,0 | High | low |
| 13 | 58 | M | 1,2 | 16,70 | 0,0 | n.a. | High | low |
| 14 | 58 | M | 2,0 | 89,60 | occasional | n.a. | High | low |
| 15 | 30 | M | 1,1 | 10,50 | 0,0 | n.a. | Low | low |
| 16 | 43 | M | 0,9 | 13,60 | 0,0 | n.a. | High | low |
| 17 | 37 | M | 13,0 | 115,70 | 4,0 | n.a. | high | High |
| 18 | 48 | M | n.a. | 204,9 | 1,0 | n.a. | high | High |
| 19 | 43 | F | n.a. | 294,00 | n.a. | n.a. | n.a. | n.a. |
| 20 | n.a. | n.a. | n.a. | 242,00 | n.a. | n.a. | n.a. | n.a. |
| 21 | n.a. | n.a. | n.a. | n.a. | n.a. | n.a. | n.a. | n.a. |
| 22 | 48 | F | n.a. | 95,50 | 2,0 | n.a. | n.a. | n.a. |
| 23 | 43 | M | n.a. | 187,00 | 3,0 | n.a. | high | High |
| 24 | 23 | M | n.a. | 88,00 | 1,0 | n.a. | high | High |

Table S2: Clinical features of CML patients for PTPRG mRNA expression analysis

| **Sex/**  **age** | **CML Staging** | **Source** | ***PTPRG/ABL1***  **%** | | **Sokal risk** | **Eutos**  **risk** | **Treatment** | **Follow up** | **Molecular Response** | **Cytogenetic** |
| --- | --- | --- | --- | --- | --- | --- | --- | --- | --- | --- |
|  |  |  | Diagnosis | Follow up |  |  |  |  |  |  |
| **M36** | Chronic phase | PB | 0.00 | 7.26 | LOW | LOW | DASATINIB | 6y | MR4 | t(9;22)( q34;q11.2) |
| **M47** | Chronic phase | PB | 0.20 | 13.30 | LOW | LOW | IMATINIB | 3,5y | MR3 | t(9;22)(q34;q11.2) + del 9q |
| **M68** | Accelerated phase | PB | 0.00 | 30.25 | HIGH | LOW | IMATINIB | 1,5y | MR3 | t(9;22)(q34;q11.2) |
| **M58** | Chronic phase | BM | 0.00 | 3.09 | INTERM | LOW | IMATINIB | 6y | MR4,5 | t(9;22)(q34;q11.2) |
| **M30** | Chronic phase | PB | 0.00 | 3.74 | INTERM | LOW | DASATINIB | 3y | MR4,5 | t(9;22)(q34;q11.2) |
| **F36** | Chronic phase | PB | 0.46 | 5.14 | LOW | LOW | IMATINIB | 15m | MR4,5 | t(9;22)(q34;q11.2) |
| **M57** | Chronic phase | PB | 0.01 | 2.02 | HIGH | HIGH | IMATINIB | 2y | MR3 | t(9;22) (q34;q11.2) |
| **M55** | Chronic phase | BM | 0.11 | 0.43 | LOW | LOW | IMATINIB | 8y | MR4 | t(9;22)(q34;q11.2) |
| **M39** | Chronic phase | PB | 0.06 | 1.66 | HIGH | HIGH | IMATINIB | 7y | MR4 | t(9;22)(q34;q11.2) |
| **F40** | Chronic phase | PB | 0.05 | 16.85 | LOW | LOW | IMATINIB | 1y | MR4,5 | t(9;22)(q34;q11.2) |
| **F53** | Chronic phase | PB | 0.09 | 1.61 | LOW | LOW | IMATINIB | 6y | MR3 | t(8;9;22)(q34;q11.2) |
| **M67** | Chronic phase | PB | 0.15 | 0.37 | HIGH | HIGH | IMATINIB | 3y | MR4,5 | t(9;22)(q34;q11.2) |
| **M56** | Chronic phase | PB | 0.36 | 3.51 | INTERM | LOW | IMATINIB | 2y | MR3 | t(9;22)(q34;q11.2) |
| **M42** | Chronic phase | PB | 0.46 | 1.53 | INTERM | LOW | IMATINIB | 1y | MR4,5 | t(9;22)(q34;q11.2) |
| **F56** | Chronic phase | BM | 0.84 | 0.30 | LOW | LOW | IMATINIB | 4y | MR3 | t(9;22)(q34;q11.2) |
| **F60** | Chronic phase | BM | 1.52 | 9.74 | INTERM | LOW | IMATINIB | 1y | MR4,5 | t(9;22)( q34;q11.2) |
| **F53** | Chronic phase | BM | 4.15 | 0.04 | LOW | LOW | IMATINIB | 2y | MR4,5 | t(9;22)(q34;q11.2) |
| **M61** | Chronic phase | PB | 0.13 | 1.16 | LOW | n.d. | NILOTINIB | 2y | MR4,5 | t(9;22)(q34;q11.2) |
| **M55** | Chronic phase | PB | 0.37 | 9.41 | LOW | n.d. | IMATINIB | 8y | MR4,5 | t(9;22)(q34;q11.2) |
| **M** | Chronic phase | PB | n.d. | 0.0968 | LOW | LOW | IMATINIB | 5m | MR4 | t(9;22)(q34;q11.2) |
| **F** | Chronic phase | PB | n.d. | 2.728 | LOW | LOW | IMATINIB | 3m | MR4 | t(9;22)(q34;q11.2) |
| **M73** | Chronic phase | PB | 0.00 | 0.19 | HIGH | LOW | IMATINIB | 2.5y | Failure | t(9;22)(q34;q11.2) |
| **F56** | Chronic phase | PB | 0.60 | 3.64 | INTERM | LOW | IMATINIB | 3y | MR2 | t(9;22)(q34;q11.2) |
| **M83** | Chronic phase | PB | 0.00 | 0.95 | HIGH | LOW | IMATINIB | 1y | MR2 | t(9;22)(q34;q11.2) |
| **M78** | Chronic phase | PB | 0.00 | 4.56 | LOW | LOW | IMATINIB | 1y | MR2 | t(9;22)(q34;q11.2) |
| **M55** | Chronic phase | PB | 0.00 | 16.09 | INTERM | LOW | IMATINIB | 6m | MR2 | t(9;22)(q34;q11.2) |
| **F69** | Chronic phase | PB | 0.03 | 0.44 | HIGH | LOW | IMATINIB | 2y | MR2 | t(9;22)(q34;q11.2) |
| **M19** | Chronic phase | PB | 1.22 | 0.07 | LOW | LOW | IMATINIB | 7y | MR2 | t(9;22)(q34;q11.2) |
| **M78** | MPN *BCR/ABL1* POS | PB | 1.70 | 4.39 | LOW | LOW | ONCOCARBIDE | 5m | MR2 | t(9;22)(q34;q11.2) |
| **M76** | Chronic phase | PB | 1.78 | 7.01 | HIGH | LOW | IMATINIB | 2,5y | MR2 | t(9;22)(q34;q11.2) + del14 |
| **F32** | Blastic phase | PB | 3.37 | 6.76 | HIGH | HIGH | DASATINIB | 6m | MR2 | t(9;22)(q34;q11.2) |
| **M59** | Chronic phase | PB | 0.01 | 1.41 | n.d. | n.d. | NILOTINIB | 3m | MR2 | t(9;22)(q34;q11.2) |
| **F43** | Chronic phase | PB | 0.05 | 1.12 | INTERM | n.d. | NILOTINIB | 3m | MR2 | t(9;22)(q34;q11.2) |
| **M** | Chronic phase | PB | 0.00 | n.d. | HIGH | HIGH | IMATINIB | 2m | n.d | t(9;22)(q34;q11.2) |
| **M76** | Chronic phase | PB | 0.04 | 0.18 | n.d. | n.d. | IMATINIB | 6m | Failure | t(9;22)(q34;q11.2) |
| **M** | Accelerated phase | PB | 0.0113 | n.d | HIGH | HIGH | NILOTINIB | 6m | n.d | t(9;22)(q34;q11.2) |
| **M** | Chronic phase | PB | 0.60 | n.d | HIGH | HIGH | NILOTINIB | 5m | n.d | t(9;22)(q34;q11.2) |
| **M** | Chronic phase | PB | 0.00 | 0.00 | HIGH | HIGH | DASATINIB | 3m | Failure | t(9;22)(q34;q11) |
| **F** | Chronic phase | PB | 0.04 | n.d. | INTERM | LOW | DASATINIB | 3m | Failure | t(9;22)(q34;q11.2) |
